# Identification and characterization of a HEPN-MNT family type II toxin–antitoxin in *Shewanella oneidensis*

**DOI:** 10.1111/1751-7915.12294

**Published:** 2015-06-25

**Authors:** Jianyun Yao, Yunxue Guo, Zhenshun Zeng, Xiaoxiao Liu, Fei Shi, Xiaoxue Wang

**Affiliations:** 1 Key Laboratory of Tropical Marine Bio-resources and Ecology, Guangdong Key Laboratory of Marine Materia Medica, RNAM Center for Marine Microbiology, South China Sea Institute of Oceanology, Chinese Academy of SciencesGuangzhou, 510301, China; 2 University of Chinese Academy of SciencesBeijing, 100049, China

## Abstract

Toxin–antitoxin (TA) systems are prevalent in bacteria and archaea. However, related studies in the ecologically and bioelectrochemically important strain *Shewanella oneidensis* are limited. Here, we show that SO_3166, a member of the higher eukaryotes and prokaryotes nucleotide-binding (HEPN) superfamily, strongly inhibited cell growth in *S*. *oneidensis* and *Escherichia coli*. SO_3165, a putative minimal nucleotidyltransferase (MNT), neutralized the toxicity of SO_3166. Gene *SO_3165* lies upstream of *SO_3166*, and they are co-transcribed. Moreover, the SO_3165 and SO_3166 proteins interact with each other directly *in vivo*, and antitoxin SO_3165 bound to the promoter of the TA operon and repressed its activity. Finally, the conserved Rx4-6H domain in HEPN family was identified in SO_3166. Mutating either the R or H abolished SO_3166 toxicity, confirming that Rx4-6H domain is critical for SO_3166 activity. Taken together, these results demonstrate that SO_3166 and SO_3165 in *S*. *oneidensis* form a typical type II TA pair. This TA pair plays a critical role in regulating bacterial functions because its disruption led to impaired cell motility in *S*. *oneidensis*. Thus, we demonstrated for the first time that HEPN-MNT can function as a TA system, thereby providing important insights into the understanding of the function and regulation of HEPNs and MNTs in prokaryotes.

## Introduction

Toxin–antitoxin (TA) loci are widespread among bacteria and archaea. Prokaryotic genomes contain toxin–antitoxin loci that induce cell dormancy in response to various stresses, such as phage inhibition (Pecota and Wood, 1996), global gene regulation (Wang and Wood, 2011) and tolerance to antibiotics (Lewis, 2010). This is mediated by the toxin components that target essential cellular processes, such as deoxyribonucleic acid (DNA) replication (Bernard and Couturier, 1992), messenger (m)RNA stability (Wang and Wood, 2011; Wang *et al*., 2011) and protein synthesis (Prysak *et al*., 2009). Five different types of TA systems have been characterized based on the interaction mode of the TA and the molecular nature of the antitoxin. All of the toxins are small proteins, while the antitoxins function as either small protein or RNA. In type I TA systems, the RNA antitoxin interacts with the toxin transcript and either inhibits translation of the toxin protein or induces degradation of the toxin mRNA. In type II systems, the antitoxin protein neutralizes the toxicity of the toxin through direct protein–protein binding. In type III TA systems, an RNA antitoxin directly interacts with the toxin protein. Unlike the type I to type III TA loci, the protein antitoxin of the type IV TA system does not interact with the toxin directly, but suppresses its toxicity by stabilizing its target (Masuda *et al*., 2012). And a type V designation has been proposed to involve the specific cleavage of the toxin mRNA by the antitoxin protein to prevent the translation of the toxin (Wang *et al*., 2012). The type II TA system is the most well-studied of the five types due to its abundance in bacterial genomes (Unterholzner *et al*., 2013). In this system, protein antitoxins interact with protein toxins and directly neutralize its toxicity; in turn, the labile antitoxins are easily degraded by the Lon or ClpXP proteases. At least 19 different type II TA systems have been identified and characterized in *E. coli* K12 (Yamaguchi and Inouye, 2011), including MqsR-MqsA (Brown *et al*., 2009; Kasari *et al*., 2010; Wang and Wood, 2011; Wang *et al*., 2011), RelE-RelB (Takagi *et al*., 2005; Li *et al*., 2009), YafQ-DinJ (Motiejunaite *et al*., 2007; Prysak *et al*., 2009), YoeB-YefM (Kamada and Hanaoka, 2005), MazF-MazE (Kamada *et al*., 2003; Zhang *et al*., 2005) and HipA-HipB (Correia *et al*., 2006).

Strains of the *Shewanella* genus have been isolated from diverse geographic locations and habitats, including fresh and marine water columns and sediments. These strains perform versatile metabolic reactions (Konstantinidis *et al*., 2009). *Shewanella oneidensis* MR-1 is a facultative bacterium that can survive and proliferate under both aerobic and anaerobic conditions. It is also a target of extensive research in the fields of bioelectrochemical systems and bioremediation. It is the first *Shewanella* spp. whose genome has been sequenced and thus serves as the model organism to study the functional repertoire of the *Shewanella* genus (Heidelberg *et al*., 2002). It contains a large number of mobile elements and multiple potential sites for integrase-mediated acquisition of foreign DNA, indicating that the MR-1 genome is exceptionally dynamic (Romine *et al*., 2008). Owing to the extreme diversity in phenotypic and ecological features, it is difficult to accurately define the core characteristics of the genus (Fredrickson *et al*., 2008; Wu *et al*., 2011). To date, with the exception of a recently identified HipA-HipB homologue (SO_0706-SO_0705) that is involved in biofilm formation and persistence (Theunissen *et al*., 2010; Wen *et al*., 2014), studies on the identification and functional characterization of other TA systems are largely lacking in *S. oneidensis.*

In this study, we provide evidence that two *S. oneidensis* genes (*SO_3165* and *SO_3166*) are co-transcribed and both encode small proteins. SO_3166 is a potent toxin belonging to higher eukaryotes and prokaryotes nucleotide-binding (HEPN) superfamily. The cognate antitoxin SO_3165 belonging to a putative minimal nucleotidyltransferase (MNT) functions as a DNA-binding protein that represses the expression of *SO_3165* and *SO_3166*. We demonstrate experimentally that SO_3166 and SO_3166 represent a HEPN-MNT family type II TA system that regulates cell motility and confers plasmid stability.

## Results

### SO_3166 is a potent toxin

**Figure 1:**
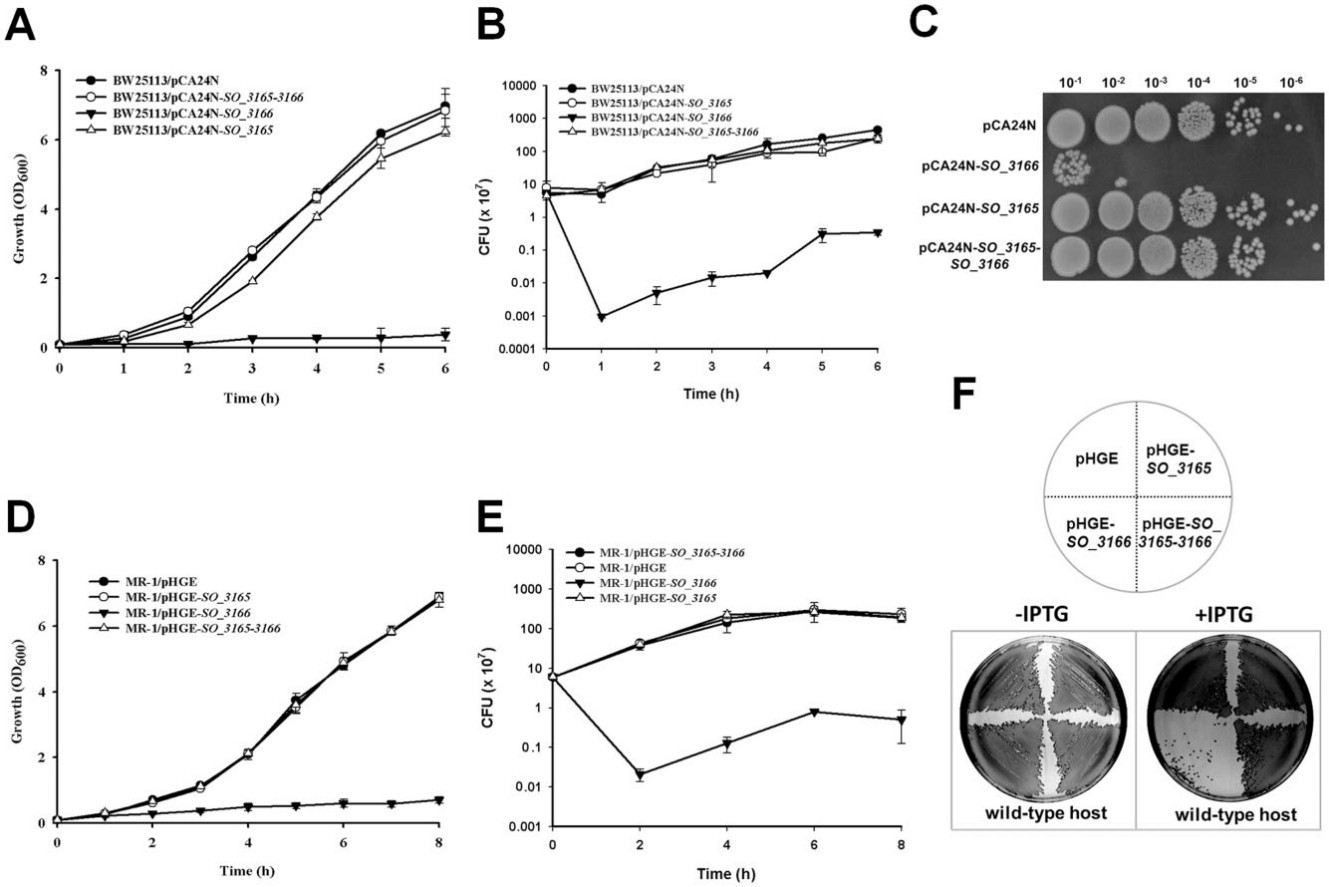
SO_3166 is the toxin and SO_3165 is the antitoxin. *E. coli* K12 BW25113 hosts containing pCA24N-based constructs were cultured in LB medium supplemented with 30 μg/μl chloramphenicol and 1 mM IPTG (added at OD_600_ of 0.1). Cell growth (A) and viability (CFU/mL) (B) were tested at the time points indicated. The cells induced for 5 h were serially diluted, dropped onto LB plates and incubated at 37°C for 16 hr (C). MR-1 hosts carrying the pHGE-based plasmids were cultured in LB with 50 μg/mL kanamycin, and 1 mM IPTG were added at OD_600_ ∼ 0.1. Cell growth (D) and viability (E) were tested at the time points indicated. (F) MR-1 hosts carrying the pHGE-based plasmids were streaked onto LB plates with 50 μg/mL kanamycin with or without 1 mM IPTG, and were incubated for 16 hr. Data are from two independent cultures, and standard deviations are shown in A, B, D and E.

The genes *SO_3166* and *SO_3165* encode small proteins that are similar in size (133 aa and 139 aa, respectively) (Fig. S1). This organization resembles a type II TA system. To probe which component of the two-gene cassette was toxic, we cloned the coding region of the two genes into the pCA24N plasmid to construct pCA24N-*SO_3165* and pCA24N-*SO_3166* (Table S1). When transformed into *E. coli* host, cells harbouring pCA24N-*SO_3166* exhibited a notable decrease in cell growth as shown by the reduction in turbidity (OD_600_) and colony forming units (CFUs). In contrast, the expression of pCA24N-*SO_3165* did not affect cell growth (Fig. 1A-C). Next, we cloned the coding region of the two genes separately into the pHGE plasmid and then conjugated the two constructs into *S. oneidensis*. Similar to the results described above, overexpressing SO_3166 greatly inhibited cell growth, while overexpressing SO_3165 did not affect cell growth (Fig. 1D-F). In addition, the overexpression of SO_3166 in *E. coli* and *S. oneidensis* did not result in cell lysis (data not shown). Corroborating these results, the production of SO_3166 in *S. oneidensis* caused a reduction in cell content without damaging the membrane and caused the cells to appear ‘swollen’ under phase contrast microscopy (Fig. S2). This result is different from the appearance of the ‘ghost’ cells caused by the overproduction of the lytic membrane toxin GhoT (Wang *et al*., 2012) and the ‘filamentous growing’ cells caused by the toxin ParE that inhibits cell division (Fiebig *et al*., 2010; Chan *et al*., 2014). Therefore, SO_3166 is a potent bacteriostatic toxin, and SO_3165 is not toxic.

### SO_3165 neutralizes the toxicity of SO_3166

Next, we tested whether the upstream SO_3165 functions as the cognate antitoxin for SO_3166. *SO_3165* neutralized the toxic effect of SO_3166 in *E. coli* when coexpressed via the pCA24N-*SO_3165–3166* plasmid (Fig. 1A–C). Similarly, coexpressing of *SO_3165* using the plasmid pGHE-*SO_3165–3166* completely neutralized the toxicity of SO_3166 in *S. oneidensis* (Fig. 1D–F). These results demonstrate that SO_3165 can counteract the toxic effect caused by the overproduction of SO_3166 in different hosts.

### SO_3166 and SO_3165 are co-transcribed

**Figure 2:**
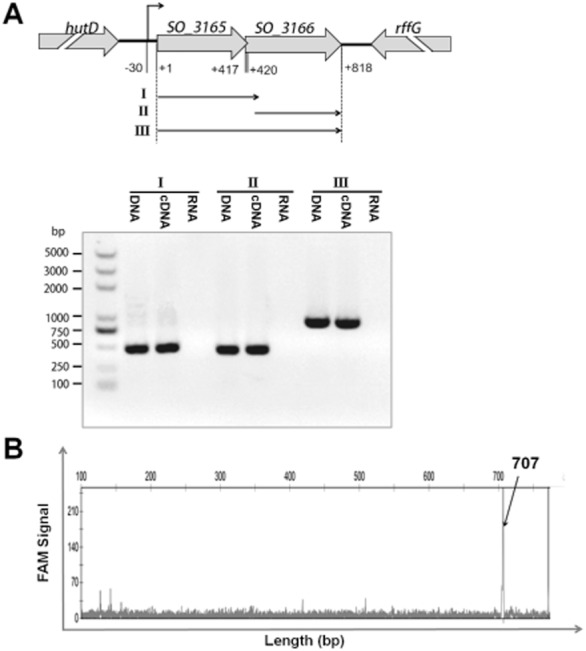
Co-transcription of *SO_3165* and *SO_3166*. (A) Primers were designed to amplify the whole open reading frame of SO_3165 (I), SO_3166 (II) and the total region that covers the start codon of SO_3165 and stop codon of SO_3166 (III). Approximately 150 ng of cDNA reverse transcribed from MR-1 RNA was used as templates to amplify the three fragments. The same amount of MR-1 genomic DNA and RNA were used as the positive and negative controls, respectively. M indicates the DNA ladder. (B) Primer extension was conducted using the 5′ FAM-labeled reverse transcriptional primer FAM-SO_3166-r and total RNA isolated from E. coli K12 BW25113 carrying the pBS(Kan)-SO_3165-3166 plasmid. The X-axis indicates the length of the cDNA with FAM and the Y-axis indicates intensity of the fluorescence signal.

The organization of the *SO_3165* and *SO_3166* genes and the impact of SO_3166 on cell growth suggested that they might compose a TA pair. *SO_3165* lies upstream of *SO_3166*, and the stop codon of the first gene and the start codon of the second gene overlap. This organization, with the antitoxin located upstream of the toxin, is a typical feature of type II TA pairs, although a few exceptions have been reported (e.g., MqsR-MqsA in *E. coli*). This organization ensures that the antitoxin is produced first and therefore is available to inactivate the toxin once it is synthesized. As shown by reverse transcription polymerase chain reaction (PCR), a single band of ∼ 800-nt was detected using a forward primer that bound to the beginning of the first gene (*SO_3165-*f) and a reverse primer that bound to the end of the second gene (Fig. S1) using complementary (c)DNA synthesized from the total RNA as template, indicating that these two genes are co-transcribed (Fig. 2A). The same size band was detected using positive control genomic DNA as a template, while no bands were obtained using the negative control total RNA as a template. To map the 5′ end of the *SO_3165–3166* operon, we performed primer extension experiment using a total of 500 nt upstream of the *SO_3165* translational start; the experiments utilized oligonucleotide FAM-SO*_3166*-r, which is complementarity to the coding region of *SO_3166* (Fig. S1). Primer extension revealed a major extension product of 707 nt in size, suggesting that the start of the transcript is located 30 nt upstream of the *SO_3165* translational start site (Fig. 2B). Therefore, *SO_3165*–*3166* is a bicistronic operon that is transcribed from a single promoter located within 30 nt of the translational start site.

### *SO_3165* and *SO_3166* form a complex in vivo

**Figure 3:**
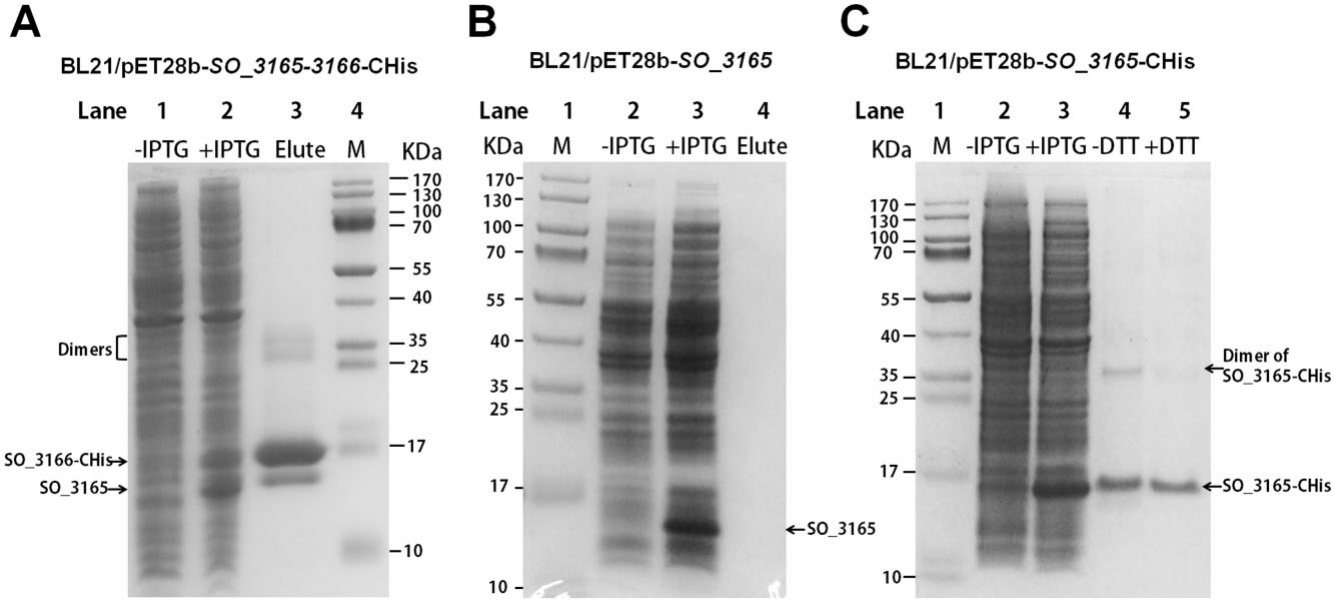
SO_3165 and SO_3166 form a complex *in vivo*. (A) Toxin SO_3166 with a C-terminal hexahistidine tag (His-tagged) was constructed together with untagged antitoxin SO_3165 to generate pET28b-*SO_3165-3166*-CHis. After induction with 1 mM IPTG, the 16.00 kDa SO_3166-CHis and a 15.57 kDa SO_3165 were induced (lane 2). The negative control was included when no IPTG was added (lane 1). During purification, SO_3166-CHis and SO_3165 were co-purified (lane 3). The protein marker (M) was loaded in lane 4. (B) SO_3165 was induced and purified via pET28b-*SO_3165* with IPTG induction under the same condition described in (A). The purified SO_3165 cannot bind to the Ni-NTA agarose beads (lane 4). (C). SO_3165-CHis (16.39 kDa) was induced and purified via pET28b-*SO_3165*-CHis with IPTG induction followed the same condition described in (A). Dimerization of SO_316 was observed (lane 4), and the addition of the reducing agent dithiothreitol (DTT) greatly reduced the dimerization (lane 5).

In type II TA systems, the toxin is normally inactivated by the formation of a protein complex between the toxin and antitoxin (Brown *et al*., 2011). Therefore, we performed a pull-down assay to determine whether SO_3165 and SO_3166 form a complex. Toxin SO_3166 with a C-terminal hexahistidine tag (His-tagged) was overexpressed together with untagged antitoxin SO_3165 via pET28b-*SO_3165–3166*-CHis. Affinity purification using nickel-nitrilotriacetic acid (Ni-NTA) agarose beads and subsequent Tricine-SDS-PAGE revealed that untagged SO_3165 could also bind to the Ni-NTA agarose beads when SO_3166-CHis and SO_3165 were co-purified (Fig. 3A). Mass spectrometry was performed to verify that the protein co-purified with SO_3166_CHis was SO_3165 (Table S2). As a negative control, untagged SO_3165 was overexpressed alone via pET28b-*SO_3165*; the overexpressed protein could not bind to the Ni-NTA agarose beads (Fig. 3B). In addition, a possible dimerization was observed when the antitoxin was expressed alone (Fig. 3C, lane 4) or coexpressed with the toxin (Fig. 3A, lane 3); the addition of the reducing agent dithiothreitol greatly reduced the dimerization of the antitoxin SO_3165 (Fig. 3C, lane 5).

### *SO_3165* represses its own promoter

**Figure 4:**
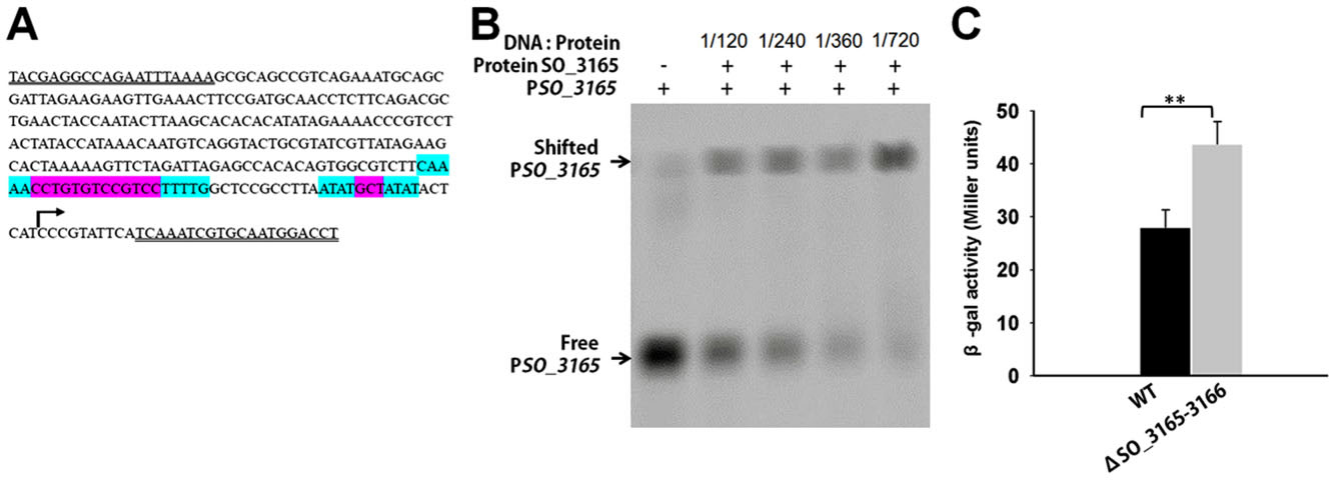
Antitoxin SO_3165 binds to the promoter of the *SO_3165–3166* operon. (A) The sequence of the promoter DNA used for EMSA (296-nt upstream of the translational start of the operon). The double underlines indicated the primers used for PCR amplification for the promoter region. The palindromic sequences are highlighted in blue, while the sequence between the two arms are highlighted in violet. (B) EMSA results demonstrating that purified SO_3165-CHis binds to the 296 bp biotin-labeled promoter DNA of *SO_3165*. The binding increases with the increasing concentrations of SO_3165-CHis protein. (C) Mid-log-phase cells of the indicated strains carrying the integrated reporter system (300-nt upstream of the translational start of the operon) were collected and tested for β-galactosidase activity. Error bars represent standard deviations for triplicate cultures. Asterisks represent a statistically significant difference between the wild-type and indicated mutants (P < 0.001; n = 3).

In typical type II TA systems, the antitoxin alone or in the context of the TA complex binds to its promoter and negatively regulates the transcription of TA. SO_3165 was predicted to belong to the MNT superfamily (Fig. S3); however, in contrast to previously identified Type II antitoxins, it does not seem to contain a predicted DNA-binding domain. To check whether SO_3165 can bind to the promoter of the TA operon, we performed electrophoresis mobility shift assays (EMSA) using purified C-terminal His-tagged antitoxin (Fig. 3C) and PCR products covering 300 nt promoter regions of the operon (Fig. 4A). SO_3165 specifically bound to its promoter region in a concentration-dependent manner (Fig. 4B). Moreover, we also conducted an *in vivo* promoter activity assay by integrating a P*_SO_3165–3166_*-*lacZ* fusion suicide plasmid into the *S. oneidensis* genome of the wild type and Δ*SO_3165–3166* strains. The promoter activity was increased 1.6 ± 0.3-fold in the Δ*SO_3165–3166* strain (Fig. 4C), suggesting that the presence of SO_3165–3166 repressed its activity. Two palindromes are located near the −10 and −35 regions (**Fig.** 4**A**); thus, repression of SO_3165 may occur through its binding to the palindromes in a similar manner to that described for the type II antitoxin MqsA.

### Key residues for determining the toxicity of *SO_3166*

**Figure 5:**
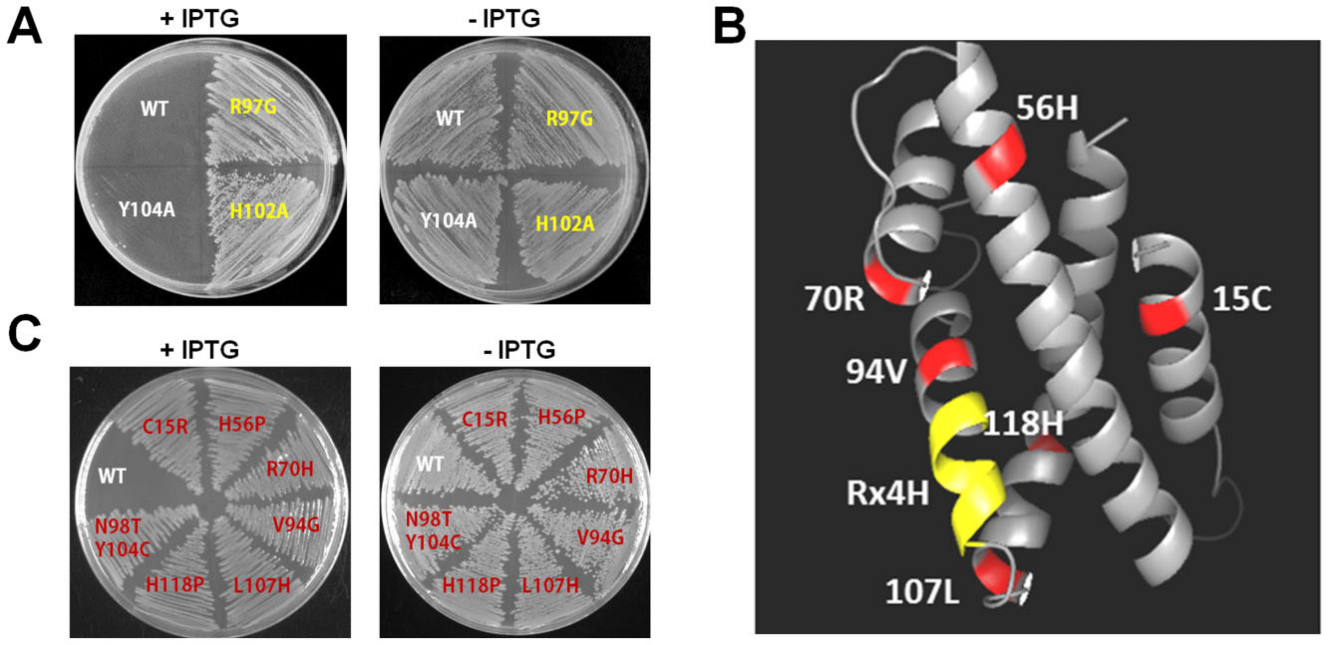
Key residues for determining SO_3166 toxicity. (A) Toxicity results of single-site mutagenesis of the R and H in Rx4H region and an adjacent tyrosine of toxin SO_3166 in the pCA24N-*SO_3166* plasmid in DH5α. (B) Predicted 3-D structure of SO_3166. The conserved domain Rx4H is situated at the end of one helix (yellow). Other residues obtained by epPCR assay that affected SO_3166 toxicity are shown in red. (C) Toxicity test of seven strains expressing different mutated SO_3166 proteins obtained by epPCR. WT indicates the wild-type SO_3166 protein; the remainders are mutated proteins. The number in the mutated protein indicates the position of the amino acid in SO_3166. Overnight cultures were streaked on 30 μg*/*mL chloramphenicol LB plates with or without 0.5 mM IPTG. Two independent cultures were evaluated for each; only one representative image is shown here.

SO_3166 was predicted to belong to HEPN superfamily (Fig. S4). The majority of HEPN domains contain a conserved Rx4-6H motif, where x is any amino acid and the residue immediately after the conserved R is typically a polar amino acid, and 4–6 indicates the number of amino acids between R and H. The conserved Rx4-6H motif has emerged as the most strongly conserved feature of the HEPN domain, and studies have suggested that the Rx4-6H motif might serve as a novel RNase active site (Anantharaman *et al*., 2013). Sequence analysis of SO_3166 revealed a conserved Rx4-6H domain (RNIAVH), with the polar amino acid N found right after the R. To investigate the importance of the conserved Rx4-6H domain in determining the toxicity of SO_3166, we performed site-directed mutagenesis on R (at position 97, from R to G) and H (at position 102, from H to A) separately. The results showed that mutation of either the R or H completely abolished the toxicity of SO_3166 (Fig. 5A). The 3D structure predicted by the Swiss-Model Server (Biasini *et al*., 2014) indicated that the conserved domain is situated at the end of one helix, probably in an open area that becomes available for catalysis following solvent exposure (Fig. 5B). In contrast, mutation of an adjacent tyrosine (at position 104, from Y to A) did not affect the toxicity of SO_3166. In addition, error-prone PCR was performed to explore whether other residues are required for the toxicity of SO_3166 using pCA24N-*SO_3166* as template. Six mutants with single amino acid changes caused a complete loss of the toxicity of SO_3166 (positions 15, 56, 70, 94, 107 and 118). Moreover, one mutant with two amino acid changes at positions 98 and 104 also lost toxicity (Figs. 5B and C). Because the change at position 104 did not affect the toxicity of SO_3166 in the site-directed mutagenesis experiments, this result suggests that the N at position 98 located immediately downstream of the conserved R in the Rx4-6H domain is also critical for the toxic effect of SO_3166 (Fig. 5C). Taken together, these results show that three amino acids inside the Rx4-6H domain and six additional amino acids outside of the domain are critical for the toxicity of SO_3166.

### SO_3166-SO_3165 confers plasmid stability in E. coli

**Figure 6:**
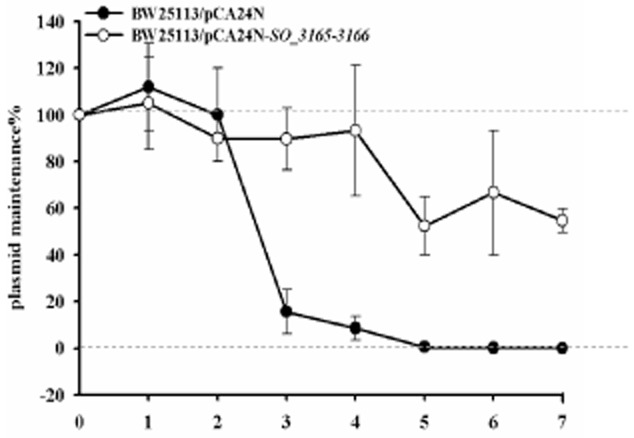
SO_3166-SO_3165 confers plasmid stability in *E*. *coli*. *E. coli* K12 BW25113 harboring plasmids pCA24N and pCA24N-*SO_3165-3166* were used for the plasmid stability assay. Overnight cultures were 1% diluted in LB medium without any antibiotics, then incubated at 37°C for 12 hr. This process was repeated every 12 hr for 7 days. Three independent cultures were conducted, and the data are shown as means ± standard deviations.

One role of TAs is to maintain extrachromosomal elements such as plasmids. In this study, plasmid pCA24N exhibited a loss of ∼ 85% from *E. coli* when grown without selection (no addition of antibiotics) for 3 days, whereas no significant plasmid loss occurred when TA pair SO_3166-SO_3165 was introduced into pCA24N. The pCA24N plasmid was completely lost from *E. coli* cells after 5 days, while more than 50% of pCA24N-*SO_3165–3166* was retained in *E. coli* cells in the absence of selection pressure from antibiotics (Fig. 6). These results suggest that SO_3166-SO_3165 provides plasmid stabilization.

### SO_3166-SO_3165 represses motility

**Figure 7:**
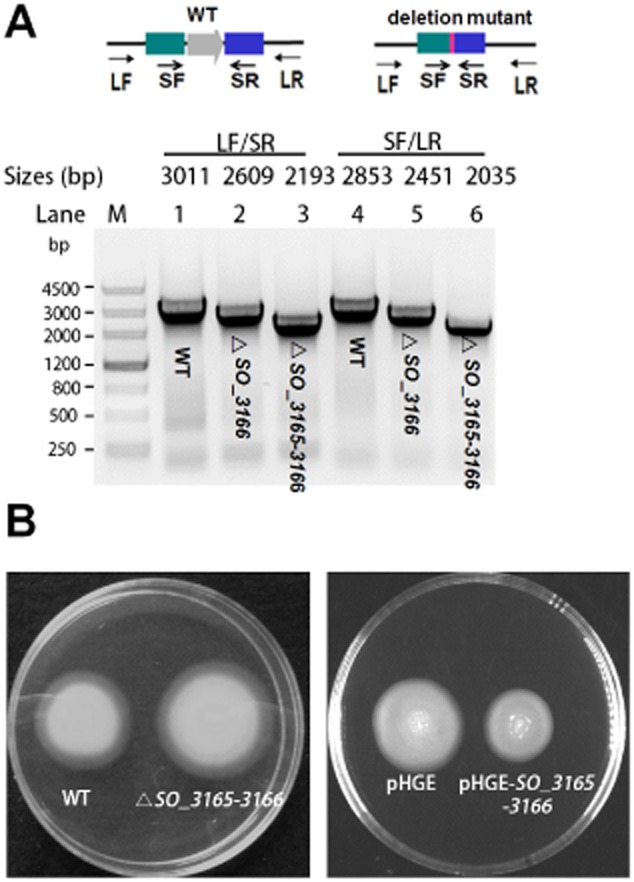
SO_3166-SO_3165 represses motility in *S*. *oneidensis*. (A) In-frame deletion mutant of the toxin gene *SO_3166* and the *SO_3165-SO_3166* operon. Primer LF indicates the long forward primer and SF indicates the short forward primer. Primers SR and LR indicate the short reverse and long reverse primers, respectively. PCR products are indicated with the expected sizes using genomic DNA from the wild-type (WT) and the deletion mutants. (B) Swimming motility test for the *SO*_*3165-3166* deletion mutant *versus* WT (left plate), and for wild-type MR-1 co-expressing *SO_3165* and *SO_3166 versus* empty vector (right plate). Two independent cultures were evaluated for each; only one representative image is shown here.

To probe the physiological function of the TA pair SO_3166-SO_3165, we constructed an in-frame deletion mutant of the toxin, antitoxin and TA pair. We successfully deleted the toxin gene and the TA pair together (Fig. 7A). However, deletion of the antitoxin gene *SO_3165* alone was not successful after several attempts. A lethal effect of deleting the antitoxin alone has been reported for other TA pairs, suggesting that chromosomal expression of the toxin could provide high toxicity to host cells without repression from the cognate antitoxin (Baba *et al*., 2006; Zhang *et al*., 2006; Goulard *et al*., 2010). Previously, the MNT family has been shown to confer resistance to aminoglycoside antibiotics such as kanamycin (Pedersen *et al*., 1995). However, there were no significant differences in cell survival following the deletion of *SO_3165–3166* when the cells were challenged with sublethal concentrations of kanamycin (2.5 μg ml^-1^), gentamycin (1 μg ml^-1^) and streptomycin (10 μg ml^-1^) (data not shown). Cell survival upon deleting *SO_3165- 3166* was also unchanged when cells were subjected to acid stress (pH 4.5 for 30 min), oxidative stress (30 mM H_2_O_2_ for 20 min) and heat stress (45°C for 10 min). Next, we tested whether the expression of the toxin and antitoxin affected swimming motility. The results showed that the co-deletion of *SO*_*3165–3166* slightly increased swimming motility. In agreement with this result, coexpression of *SO_3165* and *SO_3166* repressed swimming motility (Fig. 7B).

## Discussion

Collectively, our results strongly support the hypothesis that SO_3166-SO_3165 in *S. oneidensis* (the predicted HEPN-MNT module) forms a type II TA pair. These results are as follows: (i) both proteins are small, (ii) the two genes form an operon (*SO_3165–3166*) because they are co-transcribed and there is an overlap between the coding regions of the two genes, (iii) SO_3166 functions as a potent toxin that inhibits growth both in *S. oneidensis* host and in *E. coli* host, (iv) antitoxin SO_3165 blocks SO_3166-mediated toxicity, and SO_3165 and SO_3166 form a complex, (v) the antitoxin binds to the promoter of the *SO_3165–3166* operon and (vi) deletion of antitoxin SO_3165 in the presence of SO_3166 is lethal. These features fit a typical type II TA system, making SO_3166-3165 the second identified and characterized TA system in *S. oneidensis*.

The HEPN domain and MNT domain identified here for SO_3166 and SO_3165, respectively, have been previously suggested to a resemble Type II TA system in other systems (Makarova *et al*., 2009). The antitoxin SO_3165 was aligned to the MNT superfamily, while the toxin SO_3166 belonged to the HEPN substrate-binding superfamily of the four-helical bundle fold. Minimal nucleotidyltransferase and the accompanying subfamilies of HEPN proteins are prevalent in prokaryotic genomes. Both of these protein families have been previously described, but their biological functions have remained elusive (Makarova *et al*., 2009). Initially, MNT was predicted to act as a toxin through bioinformatics analysis because it appeared to be the only active enzyme in the HEPN-MNT module (Makarova *et al*., 2009). However, recent genome-scale surveys of toxic proteins by shotgun cloning suggested that the HEPN-containing protein should be the toxin, and the MNT-containing protein should function as the antitoxin for the HEPN-MNT module (Kimelman *et al*., 2012). Indeed, this type of system was validated by a Hhal TA pair in *Hoeflea halophia* in which HhalT with the HEPN domain was the toxin (Kimelman *et al*., 2012). Here, we specifically demonstrated that the HEPN-domain containing protein SO_3166 was toxic, while the MNT-domain containing protein was not toxic. The toxicity tests of these two proteins were confirmed not only in the *E. coli* host, but also in its original host. Moreover, previous studies showed that the HEPN and MNT families can combine with each other and form 2:2 heterotetramers (Lehmann *et al*., 2005). This finding is in agreement with our study, where we found both direct protein–protein interactions between SO_3165 and SO_3166 and dimerization of these proteins. To the best of our knowledge, this is the first study to provide experimental evidence showing that HEPN-MNT can function as a type II TA system via a point-by-point validation of the common features of type II TA systems.

The major role of toxins that target nucleic acids in physiological conflicts of all types are important for its host (Anantharaman *et al*., 2013). Based on comparative genomics analysis, HEPN domain-containing nucleases are the most common immunity-associated toxins (Makarova *et al*., 2013) and are essential components of numerous toxin–antitoxin abortive infection systems. These systems are tightly associated with many restriction-modification and CRISPR-Cas systems. Thus, toxins with HEPN domains play important roles in the adaptation to different stresses. A recent bioinformatics analysis suggested that some of the HEPN superfamily members correspond to the previously biochemically characterized catalytic domain of RNase (Anantharaman *et al*., 2013). Rx4-6H is the most conserved domain in HEPN, and site-directed mutagenesis of Rx4-6H in several HEPN domain-associated proteins both can abolish their activity (Anantharaman *et al*., 2013). For example, the histidine corresponding to the conserved H in the Rx4H motif is essential for the nuclease activities of the kinase-extension nuclease (KEN) domain of RNase L and the RNase domains of RloC and PrrC (Davidov and Kaufmann, 2008; Lee *et al*., 2008; Meineke and Shuman, 2012). In this study, we showed that R and H are both important for determining the toxic effect of SO_3166, suggesting that SO_3166 may have nuclease activity.

‘Swollen’ morphology caused by overproduction of SO_3166 appears different from the cell morphology caused by overproduction of toxin MqsR with endoribonuclease activities which makes cells appear more condensed (Wang *et al*., 2013), suggesting that the cellular targets of SO_3166 and MqsR are different. For type II toxins in *E. coli*, MqsR and MazF have been shown to exhibit sequence-specific mRNA cleavage which is independent of translation (Zhang *et al*., 2003; Yamaguchi *et al*., 2009; Vesper *et al*., 2011), while RelE, HigB, YafQ and YoeB have been shown to cleave RNA in a ribosome-dependent manner (Christensen and Gerdes, 2003; Pedersen *et al*., 2003; Hurley and Woychik, 2009; Neubauer *et al*., 2009; Prysak *et al*., 2009; Zhang and Inouye, 2009; Christensen-Dalsgaard *et al*., 2010). Recent studies have also shown that there are different targets (mRNAs or transfer (t)RNA) for VapC family toxins in different bacterial hosts (Winther and Gerdes, 2011; 2012; McKenzie *et al*., 2012). The effect of the TA pair SO_3166-SO_3165 on motility may be due to the differential decay of flagella-related mRNAs by SO_3166. We attempted to purify the SO_3166 protein, but high expression of the toxin alone was nearly impossible, as observed in other TA systems (Fico and Mahillon, 2006; Zhang *et al*., 2006; Goulard *et al*., 2010). Furthermore, it was hard to obtain the wild toxin with high purity when co-purified with its cognate antitoxin SO_3165 due to the relatively tight protein–protein interactions between them (Fig. 3A). Analysis of purified SO_3166 via *in vivo* studies is thus still warranted to elucidate the biochemical function of SO_3166 and to identify the cellular targets of SO_3166.

In this study, we showed that the MNT domain-containing antitoxin SO_3165 possesses a DNA-binding function. This is the first report showing that the MNT antitoxin can bind to the promoter of the TA operon. Unlike the well-studied *mqsRA* promoter which has two highly similar palindromes, the two palindromes of *SO_3165–3166* promoter are dissimilar, with one at −10 region with short intervening region, and the other at −35 with long intervening region (Fig. 4A). Makarova and colleagues (2009) proposed in 2009 that the HEPN-MNT family should be the prime target for experimental study to distinguish whether it functions as a TA or represents an antibiotic resistance system. Here, we provide evidence that the HEPN-MNT module SO_3166–SO_3165 functions as a TA system, but does not affect resistance to antibiotics. Thus, this module is not likely to function as an antibiotic inactivation system via the nucleotidylation of antibiotic molecules. However, a predicted nucleotidyltransferase shown to be a type IV toxin could function as a guanosine triphosphate (GTP)-binding nucleotidyltransferase; moreover, its toxicity could be neutralized by a novel DNA-binding antitoxin that possessed an HEPN domain (Dy *et al*., 2014). Mutation of the tyrosine (at position 104, Y to A) near the Rx4-6H domain has been suggested to link the adenylation of tyrosine by nucleotidyltranferase (Anantharaman *et al*., 2013); however, this mutation did not affect the toxicity of SO_3166 or the antitoxin activity of SO_3165 because SO_3165 could still neutralize the toxicity of SO_3166 with a mutated tyrosine at this position. Thus, nucleotidyltransferases may have a wider function than previously described.

Higher eukaryotes and prokaryotes nucleotide-binding-nucleotidyltransferase appears to be mobile given their non-uniform distribution across bacterial and archaeal genomes. Additionally, two HEPN-MNT units have been found in *Pseudomonas aeruginosa* and *Agrobacterium tumefaciens* plasmids. The physiological functions of this TA system, such as anti-phage activity, have been reported for the HEPN domain-containing toxin RnlA of the type II TA system RnlA-RnlB (Koga *et al*., 2011; Anantharaman *et al*., 2013). Although speculative, there are at least 19 type II TA systems in *E. coli* (Yamaguchi *et al*., 2009), as well as redundant TA systems in many if not most bacteria (Pandey and Gerdes, 2005) (e.g. *Mycobacterium tuberculosis* has at least 88 TA systems (Belitsky *et al*., 2011)). Thus, it is tempting to speculate that the reason for the redundancy is that each type II TA system allows the cell to respond to a specific stress or group of stresses in a highly regulated fashion. Therefore, upon a specific stress the role of each TA system may be to reduce growth and direct metabolism towards a new set of mRNAs (that are primarily not cleaved) and to create a small sub-population of persister cells. Our current knowledge tremendously exceeds the notion of plasmid stabilization that was proposed when the TA field was in its infancy (Wang and Wood, 2011). In the future, it will be interesting to pinpoint more specific purposes of TA systems in different microorganisms and to investigate their regulation and impact on the modulation of single cells or a population. The use of TA systems from different microorganisms in various fields of biology is just emerging.

## Experimental procedures

### Bacterial strains, plasmids and growth conditions

The *S. oneidensis* and *E. coli* strains and plasmids used in this study are listed in Table 1, and the sequences of all primers used in this study are listed in Table S1. The *E. coli* strains were grown in Luria-Bertani (LB) at 37°C. A total of 0.3 mM DAP (2,6-diamino-pimelic acid) was added when culturing WM3064. *Shewanella oneidensis* were grown in LB at 30°C. Chloramphenicol (30 μg ml^-1^) was used for maintaining the pCA24N plasmids, kanamycin (50 μg ml^-1^) was used for maintaining plasmids pHGE, pHGEI01 and pBS (Kan); gentamycin (15 μg ml^-1^) was used for maintaining the pHGM01 plasmid, and spectinomycin (100 μg/ml) was used for maintaining the pBBR-Cre plasmid.

**Table 1:** Bacterial strains and plasmids used in this study

Bacterial strains/Plasmids	Description^a^	Source
E. coli strains		
BL21(DE3)	F^-^ompT hsdS_B_(r_B_^-^m_B_^-^) gal dcm λ(DE3) Ω P_tacUV5_::T7 polymerase	Novagen
K12 BW25113	lacI^q^ rrnB_T14_ ΔlacZ_WJ16_ hsdR514 ΔaraBAD_AH33_ ΔrhaBAD_LD78_	(Baba *et al*., 2006)
WM3064	thrB1004 pro thi rpsL hsdS lacZΔM15 RP4-1360) Δ(araBAD)567 ΔdapA1341::[erm pir(wt)]	W. Metcalf, UIUC
DH5α	F^–^ Φ80lacZΔM15 Δ(lacZYA-argF) U169 recA1 endA1 hsdR17 (rK^–^, mK^+^) phoA supE44 λ^–^ thi-1 gyrA96 relA1	Invitrogen
S. oneidensis strains		
MR-1	Wild type	(Shi *et al*., 2013)
ΔSO_3165–3166	In frame deletion of SO_3165–3166 operon	This study
ΔSO_3166	In frame deletion of SO_3166 gene	This study
Plasmids		
pCA24N	Cm^R^; lacI^q^, IPTG inducible expression vector in E. coli	(Kitagawa *et al*., 2005)
pCA24N-SO_3165	Cm^R^; lacI^q^, P_T5-lac_:: SO_3165	This study
pCA24N-SO_3166	Cm^R^; lacI^q^, P_T5-lac_:: SO_3166	This study
pCA24N-SO_3165–3166	Cm^R^; lacI^q^, P_T5-lac_:: SO_3165–3166, IPTG inducible expression vector in E. coli	This study
pHGE	pHGE-Pt_ac_, Km^R^, IPTG inducible expression vector in S. oneidensis	(Shi *et al*., 2013)
pHGE-SO_3165	Km^R^; expression vector for SO_3165	This study
pHGE-SO_3166	Km^R^; expression vector for SO_3165	This study
pHGE-SO_3165–3166	Km^R^; expression vector for SO_3165–3166	This study
pHGM01	Gm^R^; Cm^R^; Ap^R^; sacB; Ori-R6K; suicide plasmid for generating in-frame deletions	(Shi *et al*., 2013)
pHGM01-SO_3166	pHGM01 containing the PCR fragments for deleting SO_3166	This study
pHGM01-SO_3165–3166	pHGM01 containing the PCR fragments for deleting SO_3165–3166	This study
pET28b		
pET28b-SO_3165–3166-CHis	Km^R^, lacI^q^, pET28b P_T7-lac_:: SO_3165–3166 with C-terminal His-tagged	This study
pET28b-SO_3165	Km^R^, lacI^q^, pET28b P_T7-lac_:: SO_3165	This study
pET28b-SO_3165-cHis	Km^R^, lacI^q^, pET28b P_T7-lac_:: SO_3165 with His-tagged at C-terminal	This study
pBS(Kan)	Km^R^; pBS(Kan)	(Canada *et al*., 2002)
pBS(Kan)-PSO_3165–3166	Km^R^; pBS(Kan) with 500-nt upstream and coding region of SO_3165–3166	This study
pBBR-Cre	Helper vector for removing antibiotic cassette	(Fu *et al*., 2014)
pHGEI01	Integrative lacZ reporter vector	(Fu *et al*., 2014)
pHGEI01-PSO_3165–3166	Integrative lacZ reporter vector carrying 300-nt upstream of SO_3165–3166	This study

**a**. Cm^R^, Km^R^, Gm^R^ and Ap^R^ indicate chloramphenicol, kanamycin, gentamycin and ampicillin resistance, respectively.

### In-frame deletion of the *S*. *oneidensis* toxin and antitoxin

The coding region of *SO_3166* and *SO SO_3165–3166* were deleted from *S. oneidensis* using the fusion PCR method as previously described (Jin *et al*., 2013). The primers used are listed in Table S1. Briefly, a homologous product was obtained by a three-step PCR that allowed the amplification of a linear PCR fragment. The upstream and downstream regions of the target gene open reading frame were PCR amplified from wild-type genomic DNA and subsequently joined through fusion PCR via a complementary ‘linker’ region that was added to the 5′ end of each inner primer. Then, the products were ligated into the suicide plasmid pHGM01 by site-specific recombination and transformed into *E. coli* strain WM3064. This fusion product was inserted into the cloning vector pHGM01-sacB by homologous recombination. Integration of the recombinant plasmid into the chromosome was selected by gentamycin resistance and confirmed by PCR. Verified transconjugants were grown in LB without NaCl, and plated on LB supplemented with 10% sucrose. Gentamycin-sensitive and sucrose-resistant colonies were screened by PCR. The regions near the deletions were verified by PCR followed by DNA sequencing.

### RNA isolation and RT-PCR

Bacteria in the exponential phase were pelleted by centrifugation at 2500 × g for 2 min. Total RNA was isolated using the QIAGEN RNase Mini kit (Valencia, CA, USA) as described previously (Ren *et al*., 2004). To avoid DNA contamination, the total RNA was treated with 20 U of DNase for 30 min during the isolation process. The cDNA synthesis was conducted using the reverse transcription system (Promega, Madison, WI) according to the instruction and operation manual.

### Primer extension

The 5′ end FAM dye (6-carboxyfluorescein)-labeled primer FAM-*SO_3166*-r (Table S1) was ordered from Invitrogen. A total of 30 μg of total RNA was added to 2 × 10^−4^ pmol of 5′ end-labeled primer, and the mixture was added to 3 μl of 10 × first-strand and 37.5 U AMV reverse transcriptase (Promega). The RNA mix was annealed to the primers by incubating at 37°C for 1 h, and the products were concentrated with centrifugal filter units (Millipore, USA). The products were screened with an ABI3730 DNA Analyzer (Applied Biosystems, USA), and the results were analysed using Genemapper (Version 4.1). Ribonucleic acid was isolated from *E. coli* BW25113/pBS (Kan)-P*SO_3165–3166* (including 500-nt upstream of the translational start and the coding region of *SO_3165–3166*) with its own promoter.

### Protein expression and purification

SO_3165 and SO_3165-SO_3166 each containing a six histidine tag at the C-terminus and SO_3165 with no tag were purified via BL21 (DE3) with pET28b-*SO_3165*-CHis, pET28b-*SO_3165–3166*-CHis and pET28b-*SO_3165* respectively. The strains were induced with 1 mM IPTG at a turbidity of 0.1 for 6 h. Then, the cells were collected and re-suspended in 10 ml of lysis buffer [50 mM monosodium phosphate buffer (pH 8.0), 300 mM NaCl, 5 mM imidazole and protease inhibitor cocktail (Sigma-Aldrich, USA)]. The samples were lysed with the FastPrep-24 tissue and cell homogenizer five times for 20 s. Ni-NTA agarose beads were used according to the manufacturer's protocol. Purified proteins were desalted using a desalination column with 20 mM Tris-HCl buffer (pH 8.0), and the protein concentration was measured using a Bi Yuntian BCA assay kit (Haimen, China). Tricine-SDS-PAGE was performed as previously described (Schägger, 2006). A total of 25 μg of protein from each sample was loaded for SDS-PAGE.

### EMSA

Electrophoretic mobility shift assays were performed as previously described (Kim *et al*., 2010). The promoter region of the *SO_3165–3166* operon (296 nt) (P*SO_3165–3166*) was amplified by PCR from genomic DNA using the Pfu DNA polymerase from genomic DNA with primers P*SO_3165*-f and P*SO_3165-*r. The PCR products were gel purified with a QIAquick Gel Extraction Kit (Qiagen) and labeled with biotin using the Biotin 3′-end DNA Labeling Kit (Pierce). For the binding reactions, biotin-P*SO_3165–3166* (0.05 pmol) DNA was incubated with purified SO_3165-CHis protein for 1 h at room temperature. The binding reaction conditions were performed with the non-specific competitor DNA (poly dI-dC) and NP-40 in buffer containing 10 mM HEPES (pH 7.3), 20 mM KCl, 1 mM MgCl_2_, and 5% glycerol at 25°C for 1 h. The samples were run on a 6% DNA retardation gel (Invitrogen) at 100 V in 0.5 × TBE (10 mM Tris borate at pH 8.3 and 2 mM EDTA) for 90 min. Then, the DNA was transferred to a nylon membrane at 390 mA for 45 min, followed by UV cross-linking at 302 nm. Chemiluminescence was performed with the LightShift Chemiluminescent EMSA Kit (Thermo Scientific, Rockford, IL) according to the manufacturer's protocol.

### Stress assays

For stress assays, the cells were grown to a final OD_600_ close to 1.0 and diluted 10^1^ to 10^7^ via 10-fold serial dilution in 0.85% NaCl solution. The dilutions were plated onto LB agar with different stressors to determine cell viability (Donegan *et al*., 1991). The sublethal concentrations of antibiotics used included 2.5 μg ml^-1^ kanamycin, 1 μg ml^-1^ gentamycin and 10 μg ml^-1^ streptomycin. Oxidative, heat and acid stress treatments were conducted by incubation with 30 mM H_2_O_2_ for 20 min, at 45°C for 10 min and at pH 4.5 for 30 min respectively.

### Promoter activity assay

Deoxyribonucleic acid fragments 300 nt upstream of the translational start of S*O_3165* were generated by PCR, digested with *EcoR*I and *BamH*I and cloned into the promoter-less *lacZ*-fusion vector pHGEI01 (Fu *et al*., 2013) to create plasmid pHGEI01-*PSO_3165–3166*. The resulting plasmid was verified by sequencing and introduced into *S. oneidensis* strains for integration. The antibiotic marker was subsequently removed using plasmid pBBR-Cre following the previously described protocol (Wu *et al*., 2011; Fu *et al*., 2014). Mid-log phase (OD_600_ ∼ 0.7) cells of the indicated strains carrying the integrated reporter system were collected by centrifugation and washed with phosphate buffered saline. The cell soluble protein and beta-galactosidase activity were determined using previously described protocols (Wu *et al*., 2011).

### Site-directed mutagenesis

Single site-directed mutagenesis (Wang and Wood, 2011; Wang *et al*., 2011) was used to mutate the Rx4H region of SO_3166. Mutation of R (CGA) to G (GGA) used primer pair SO_3166-R97G-f/-r, H (CAT) to A (GCT) used primer pair SO_3166-H102A-f/-r and Y (TAC) to A (GCC) used primer pair SO_3166-Y104A-f/-r respectively (Table S1). The mutations were verified by DNA sequencing using primers pCA24N-f and pCA24N-r.

### Error-prone PCR

Error-prone PCR (epPCR) was conducted on plasmid pCA24N-*SO_3166* using primers epPCR-f and epPCR-r as described previously (Fishman *et al*., 2004). The epPCR program was as follows: 94°C for 5 min, 30 cycles of 1 min at 94°C, 1 min at 55°C and 2 min at 72°C, followed by 10 min at 72°C for the final extension. The error rate was maintained at 18% by adjusting the concentration of MgCl_2_ to 2.5 mM and MnCl_2_ to 1 mM. The PCR products were gel purified and digested using *BseR*I and *Hind*III prior to ligation into pCA24N. The ligation mixture was transformed into *E. coli* DH5α.

### Plasmid stabilization assay

Overnight cultures of *E. coli* BW25113 carrying the plasmids pCA24N and pCA24N-*SO_3165-SO_3166* were obtained with antibiotic selection. The cultures were diluted 1% in LB medium without antibiotics, and then incubated at 37°C for 12 h. This process was repeated every 12 h. The cultures were serially diluted 10^0^–10^7^ by 10-fold from days 1 to 7, and 10 μL was dropped onto LB plates with and without 30 μg ml^-1^ of chloramphenicol. The plates were incubated at 37°C for 16 h, and then the CFU were analysed. The CFU assay was conducted every day up to 7 days.

### Swimming motility assay

Cell motility assay was performed as described previously (Wang and Wood, 2011; Wang *et al*., 2011). In brief, motility agar plates with 1% trypton, 0.25% NaCl and 0.3% agar were prepared, 50 μg ml^-1^ of kanamycin were also added to the strains containing pHGE-based constructs. About 1 μL of culture was dropped on the plates and cultured at 30°C for 24 h.

## Supporting Information

**Fig. S1.** Gene organization of *SO_3165* and *SO_3166* in *S. oneidensis*. **Fig. S2.** Cell morphology examined by phase-contrast microscopy. **Fig. S3.** SO_3165 in *S. oneidensis* belongs to the MNT substrate-binding superfamily. **Fig. S4.** SO_3166 in *S. oneidensis* belongs to the HEPN superfamily. **Table S1.** Oligonucleotides used for plasmid construction gene knockout, site-directed mutagenesis (target mutated nucleotides are in red font) and DNA sequencing. Restriction enzyme sites are underlined. f indicates forward primer and r indicates reverse primer. **Table S2.** Mass spectrometry results of the co-purified protein with SO_3166-CHis. Peptide fragments observed were highlighted in different colours, and their loci in SO_3165 protein were also shown.
