# Supplementary material for: Identification and characterization of a HEPN-MNT family type II toxin–antitoxin in Shewanella oneidensis
Source: Microb Biotechnol. 2015 Jun 25;8(6):961–73. doi: 10.1111/1751-7915.12294 (PMC4621449; doi:10.1111/1751-7915.12294)
Supplement: Supplementary file 1 — Fig. S1. Gene organization of SO_3165 and SO_3166 in S. oneidensis. Fig. S2. Cell morphology examined by phase-contrast microscopy. Fig. S3. SO_3165 in S. oneidensis belongs to the MNT substrate-binding superfamily. Fig. S4. SO_3166 in S. oneidensis belongs to the HEPN superfamily. Table S1. Oligonucleotides used for plasmid construction gene knockout, site-directed mutagenesis (target mutated nucleotides are in red font) and DNA sequencing. Restriction enzyme sites are underlined. f indicates forward primer and r indicates reverse primer. Table S2. Mass spectrometry results of the co-purified protein with SO_3166-CHis. Peptide fragments observed were highlighted in different colours, and their loci in SO_3165 protein were also shown. [file mbt20008-0961-sd1.pdf]

SUPPLEMENTARY FILE

Identification and Characterization of a HEPN-MNT Family Type II Toxin-Antitoxin in *Shewanella oneidensis*

Jianyun Yao[1,2†], Yunxue Guo[1†], Zhenshun Zeng[1,2], Xiaoxiao Liu[1], Fei Shi[1], Xiaoxue Wang[1*]

[1]Key Laboratory of Tropical Marine Bio-resources and Ecology, Guangdong Key Laboratory of Marine Materia Medica, RNAM Center for Marine Microbiology, South China Sea Institute of Oceanology, Chinese Academy of Sciences, Guangzhou 510301, PR China,

[2]University of Chinese Academy of Sciences**,** Beijing 100049, China

† These authors contributed equally to this work.

*To whom correspondence should be addressed. Tel: +86 20 89267515; Fax: +86 20 89235490; Email: xxwang@scsio.ac.cn

Keywords: Toxin-antitoxin, MNT, HEPN, *Shewanella oneidensis*

Running head: a HEPN-MNT Family Type II TA in *S. oneidensis*

Table S1. Oligonucleotides used for plasmid construction gene knockout, site-directed mutagenesis (target mutated nucleotides are in red font) and DNA sequencing. Restriction enzyme sites are underlined. f indicates forward primer and r indicates reverse primer.

**Purpose/Name**	**Sequence (5'-3')**
**Plasmid construction**	
pHEG-*SO_3165*-f	GGAATTCATGCAACAACTAAATGAAAATA
pHEG-*SO_3165*-r	CCCAAGCTTTCAAAGTGACTCCCCCCTATGAG
pHEG-*SO_3166*-f	GGAATTCATGACATTATTATCAATAAAATA
pHEG-*SO_3166*-r	CCCAAGCTTTCACTCTGCTTTTATCACATCAA
pCA24N-*SO_3165*-f	GCCCAACAACTAAATGAAAATAA
pCA24N-*SO_3165*-r	CCAAGTGACTCCCCCCTATGAGC
pCA24N-*SO_3166*-f	GCCAATGACATTATTATCAATAA
pCA24N-*SO_3166*-r	CCCTCTGCTTTTATCACATCAAT
pET28b-*SO_3165*-f	CTAGCCATGGGCATGCAACAACTAAATGAAAATAA
pET28b-*SO_3165*-r	CCCAAGCTTCTAGTGGTGGTGGTGGTGGTGAAGTGACTCCCCCCTATGAGCTTTGGC
pET28b-*SO_3166*-f	CTAGCCATGGGCATGAATGACATTATTATCAATAA
pET28b-*SO_3166*-r	CCCAAGCTTCTAGTGGTGGTGGTGGTGGTGCTCTGCTTTTATCACATCAATAAATTG
pBS(Kan)-*SO_3165*-500 up-f	GTCTAGGGTACCAGTACCGCGCGTATATTGAGCG
pBS(Kan)-*SO_3166*-r	GAGCACGAGCTCTCACTCTGCTTTTATCACATCA
FAM-*SO_3166*-r	CGAGAGTGGCTGAGTAATAA
pHGR03-$P_{so\_3165\text{-}3166}$-f	CCGGAATTC TGCCTACGAGGCCAGAATTTAAA
pHGR03-$P_{so\_3165\text{-}3166}$-r	CGCGGATCCGTAATCATGGTCATAGGTCCATTGCACGATTTGA
**PCR and DNA sequencing**	
pCA24N-f	GATAACAATTTCACACAGAATT
pCA24N-r	GTCAGAGGTTTTCACCGTCATCA

PHEG-f	CACCTCGCTAACGGATTCACC
PHEG-r	CCAATACGCAAACCGCCTC
pET28b-f	TAATACGACTCACTATAGGG
pET28b-r	TATGCTAGTTATTGCTCAG
pBS(kan)-f	GCCTTTGAGTGAGCTGATACCGCTC
pBS(kan)-r	GTGATGGTTCACGTAGTGGGCCATCG
CRSID-F	GATGTTGAAGTGGCGAGC
CRSID-V	ATGGATGCAAGCAAATGC
P*SO_3165*-f	TACGAGGCCAGAATTTAAAA
P*SO_3165*-r	AGGTCCATTGCACGATTTGA
**Gene knockout**	
delta *SO_3166*-5-O	GGGGACAAGTTTGTACAAAAAAGCAGGCTCGAACGCTCCGCTTACAGATA
delta *SO_3166*-5-I	GGTCCGGGTTCGCTATCTATTTCAAAGTGACTCCCCCTATGAG
delta *SO_3166*-3-O	GGGACCACTTTGTACAAGAAAGCTGGGTTATGCGAGCCATCCTGAATC
delta *SO_3166*-3-I	ATAGATAGCGAACCCGGACCAGATGTTGACAGAACAAGAAACG
delta *SO_3165-3166*-5-O	GGGGACAAGTTTGTACAAAAAAGCAGGCTCGAACGCTCCGCTTACAGATA
delta *SO_3165-3166*-5-I	GGTCCGGGTTCGCTATCTATTAGGTCCATTGCACGATTTGATGAATA
delta *SO_3165-3166*-3-O	GGGACCACTTTGTACAAGAAAGCTGGGTTATGCGAGCCATCCTGAATC
delta *SO_3165-3166*-3-I	ATAGATAGCGAACCCGGACCAGATGTTGACAGAACAAGAAACG
delta *SO_3166*-l-f	GACTGGGATAGTAACGGCTCAAA
delta *SO_3166*-s-f	ATCCGCTATCAAGAATGCGAATC
delta *SO_3166*-l-r	GCTTTGGTCCGTATGTTGATTGA
delta *SO_3166*-s-r	ACTTTAGGGCAAGCACAACAGCG
**Single amino acid substitution**	
SO_3166 R97G-f	GAAAATGGTCGGCCTG**G**GAAACATCGCAGTACA

SO_3166 R97G-r	TGTACTGCGATGTTTCCCAGGCCGACCATTTTC
SO_3166 H102A-f	TTGAGTTCTTGGTAGTCA**GC**TACTGCGATGTTTCGCAGGC
SO_3166 H102A-r	GCCTGCGAAACATCGCAGTA**GC**TGACTACCAAGAACTCAA
SO_3166 Y104A-f	TATCGAGGTTGAGTTCTTGG**GC**GTCATGTACTGCGATGTTTC
SO_3166 Y104A-r	GAAACATCGCAGTACATGAC**GC**CCAAGAACTCAACCTCGATA

Table S2. Mass spectrometry results of the co-purified protein with SO_3166-CHis. Peptide fragments observed were highlighted in different colors, and their loci in SO_3165 protein were also shown.

Reference<br>Scan(s)	Peptide	MH+	z	P (pro)<br>P (pep)	Score<br>XC	Coverage<br>DeltaCn	MW<br>Sp	Accession<br>RSp	Peptide (Hits)<br>Ions	Count
5542	K.TISM*YQHLQAER.Q	1492.72129	2	2.56E-04	3.72	0.46	654.5	1	17/22	2
5745	R.QAIIDDVM*ANTAAK.A	1476.73627	2	2.15E-03	3.19	0.54	507.0	1	20/26	2
8253	K.QLWGTQQDDELFAVK.T	1777.87554	2	4.56E-06	4.73	0.66	1445.2	1	21/28	2
8447	R.NSDIDIAVLAADTLDNIAR.W	2000.02947	2	2.29E-04	2.81	0.54	451.9	1	16/36	2
9743	K.LASALDSDVDLVDLRSASTV LCQQVVTQGK.Q	3188.64677	3	1.55E-10	6.77	0.73	1913.4	1	40/116	2
9932	K.LLRDNIPKLQLIYLFGSYSQG TQHR.N	2960.61049	3	2.22E-15	6.72	0.68	2843.9	1	43/96	2

toxin-antitoxin system antidote Mnt family (SO_3165) [*Shewanella oneidensis*]

```
             1        10        20        30        40        50        60
SO_3165:     MQQLNENKIIKLLRDNIPKLQLIYLFGSYSQGTQHRNSDIDIAVLAADTLDNIARWELAQ
Identified:            KLLRDNIPKLQLIYLFGSYSQGTQHRNSDIDIAVLAADTLDNIARW

             61       70        80        90        100       110    117
SO_3165:     KLASALDSDVDLVDLRSASTVLCQQVVTQGKQLWGTQQDDELFAVKTISMYQHLQAE
Identified:  KLASALDSDVDLVDLRSASTVLCQQVVTQGKQLWGTQQDDELFAVKTISMYQHLQAE

             118       128        139
SO_3165:     RQAIIDDVMANTAAKAHRGESL
Identified:  RQAIIDDVMANTAAKA
```

Figure S1. Gene organization of *SO_3165* and *SO_3166* in *S. oneidensis*. Transcriptional start site C (-30 bp upstream of the start codon of *SO_3165*) for the operon is indicated with an arrow. The sequence encoding *SO_3165* is shown in orange letters and the sequence encoding *SO_3166* is shown in blue letters. The start and stop codons for *SO_3166* and *SO_3165* are highlighted in green and square frames, respectively. The primer used for primer extension experiment is underlined.

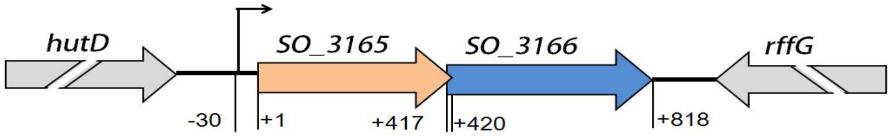

CATCCCGTATTCATCAAATCGTGCAATGGACCT

ATGCAACAACTAAATGAAAATAAAATCATAAAGTTACTGCGAG
ACAACATCCCAAAGTTGCAATTAATCTACCTGTTTGGCTCTTAT
TCGCAGGGAACACAACACCGCAACAGCGATATCGATATCGCGG
TACTCGCCGCTGACACTTTAGACAATATTGCACGCTGGGAACT
TGCCCAAAAACTCGCTTCGGCGTTGGACTCAGATGTTGATCTG
GTTGATTTACGCTCAGCATCCACCGTGTTATGCCAACAGGTAGT
CACTCAAGGGAAGCAACTTTGGGGAACACAACAAGATGATGA
ACTTTTTGCCGTAAAGACCATCTCAATGTACCAACATCTACAAG
CCGAAAGACAGGCAATTATTGATGATGTAATGGCCAACACCGC

SO_3165

AGCCAAAGCTCATAGGGGGGAGTCACTTTGAATGACATTATTA
TCAATAAAATAGCCACCATCAAACGCTGTATAAAGCGCATCCA
ACAAGTCTATGGTGATGGCAGCCAGTTCAAACAAGACTTCACC
TTGCAAGATAGCGTTATCCTCAATTTACAACGCTGCTGCGAAGC
CTGTATCGACATAGCCAATCATATTAACCGCCAACAACAACTAG
GCATCCCCCAAAGTAGCCGTGATAGCTTTACATTACTCGCACAA
AATAACC<u>TTATTACTCAGCCACTCTCG</u>GATAACTTAAAGAAAAT
GGTCGGCCTGCGAAACATCGCAGTACATGACTACCAAGAACTC
AACCTCGATATCGTTGTGCATGTTGTACAGCATCATCTTGAGGA
TTTTGAGCAATTTATTGATGTGATAAAAGCAGAGTGA

SO_3166

Figure S2. Cell morphology examined by phase-contrast microscopy. Morphology of cells overproducing SO_3166 (MR-1/pHGE-*SO_3166*) vs. cells carrying empty plasmid (MR-1/pHGE) at 0 h and 10 h. Red arrows point to the "swollen" cells. Cells were grown in LB until the OD reached ~0.1. A concentration of 1 mM IPTG was added to overexpress *SO_3166* in *S. oneidensis*. Two independent cultures were used for each strain; only representative images are shown here. Scale bars indicate 10 μm.

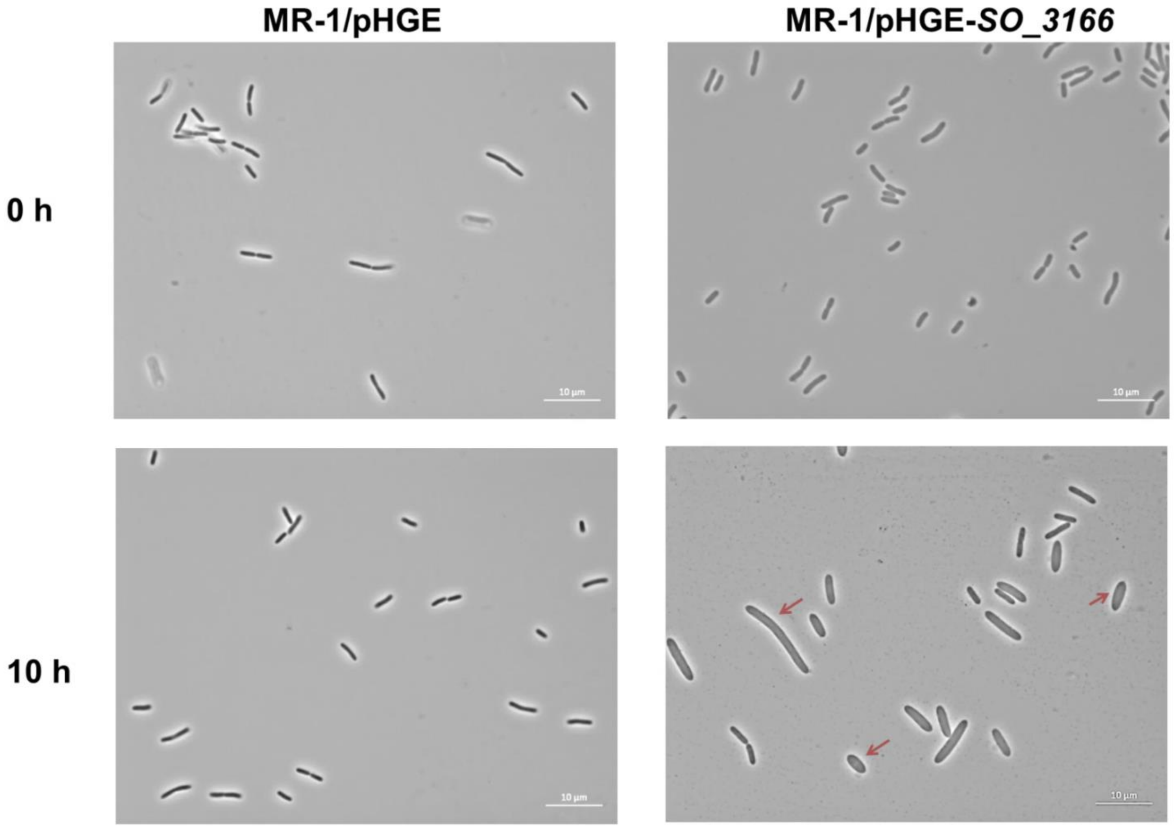

Figure S3. SO_3165 in *S. oneidensis* belongs to the MNT substrate-binding superfamily. The secondary structure of SO_3165 was predicted using the online PHYRE2 server (Kelley and Sternberg, 2009). The α-helices and β-sheets are shown. SO_3165 was predicted to have a similar secondary structure with the known secondary structure of nucleotidyltransferase 2 (np_343093.1) from *Sulfolobus solfataricus*. The MNT residues in the two proteins are boxed.

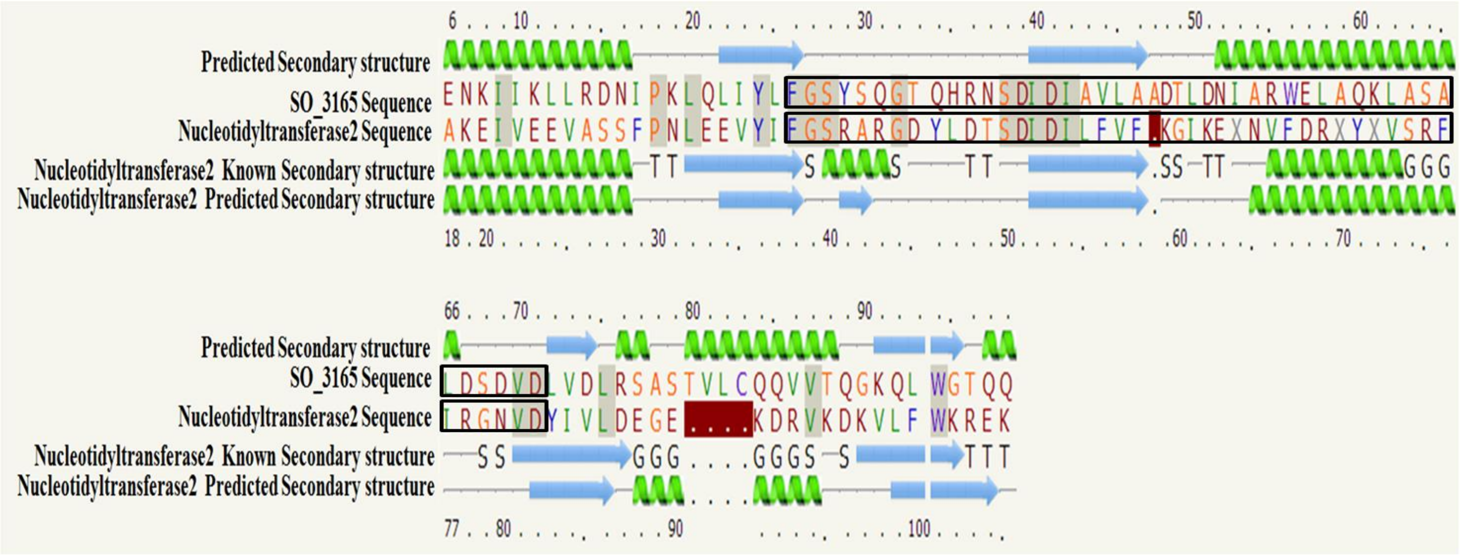

Figure S4. SO_3166 in *S. oneidensis* belongs to the HEPN superfamily. The secondary structure of SO_3166 was predicted using the online PHYRE2 server (Kelley and Sternberg, 2009). The α-helices and β-sheets are shown. The toxin SO_3166 has a similar secondary structure with the known secondary structure of the functional unknown HEPN family protein Yute 2 from *Bacillus Subtilis*. The HEPN residues in the two proteins are boxed, and conserved Rx4-6H motifs in the two proteins are also shown.

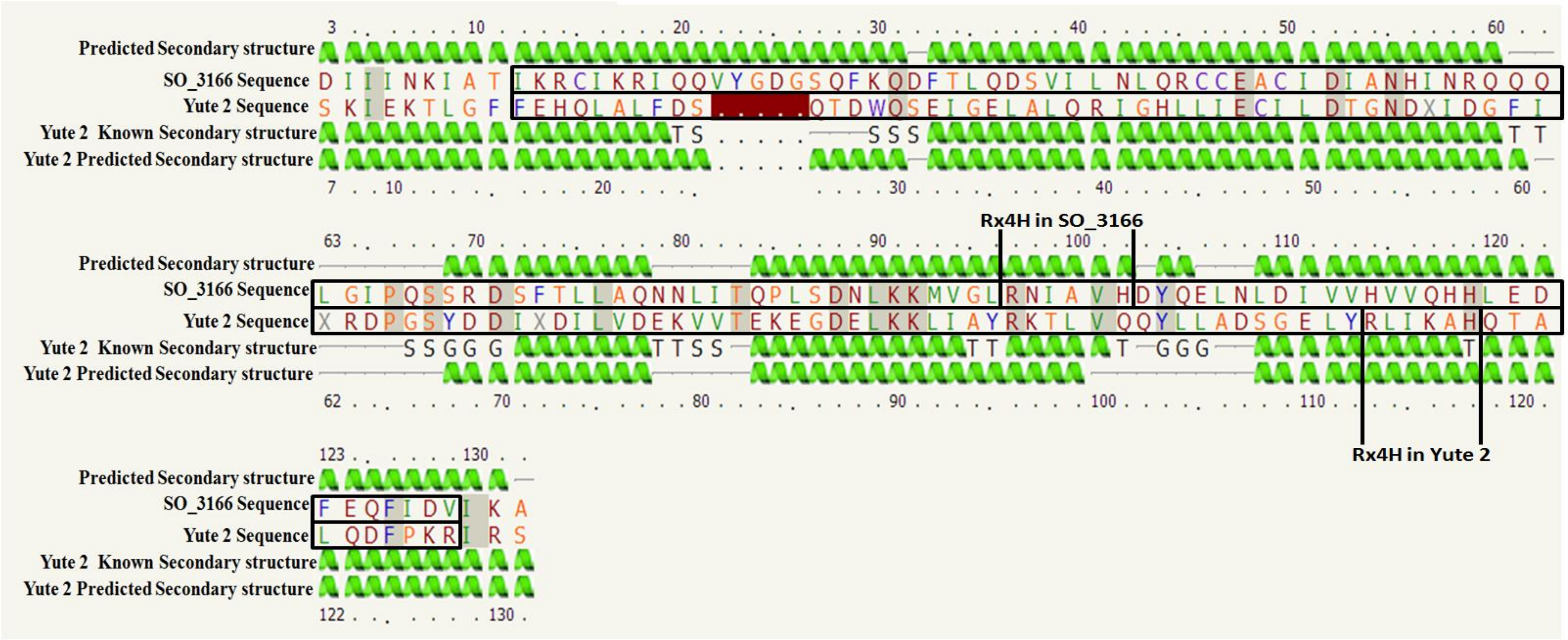

Reference:

Kelley, L.A., and Sternberg, M.J. (2009) Protein structure prediction on the Web: a case study using the Phyre server. *Nat Protoc* **4**: 363-371.
